# Xylazine-Induced Necrotic Skin Ulcers in a Fentanyl-Injecting Individual in South Florida, United States: A Case Report

**DOI:** 10.7759/cureus.66609

**Published:** 2024-08-10

**Authors:** Jesus Cervantes, Jordan Khorsandi, Kajal S Patel, Chenet Torrilus, Guillermo Izquierdo, David Serota

**Affiliations:** 1 Internal Medicine, Florida International University, Herbert Wertheim College of Medicine, Miami, USA; 2 Internal Medicine, Jackson Memorial Hospital, Miami, USA; 3 Infectious Diseases, Jackson Memorial Hospital, Miami, USA

**Keywords:** tranq-dope, tranq, fentanyl, necrotic ulcer, xylazine-induced skin ulcer, xylazine

## Abstract

Xylazine, commonly referred to as “Tranq,” is an alpha-2-adrenergic receptor agonist that is FDA-approved only as a sedative and tranquilizer for veterinary use. However, its use as an adulterant in various illicit drugs, including fentanyl, has been on the rise, leading to its street name, “Tranq-Dope.” Intravenous injection use of xylazine produces distinctive skin ulceration with accompanying necrosis, which can be considered virtually pathognomonic. A 41-year-old male with polysubstance abuse disorder presented to the emergency department with severe skin ulcerations on the right forearm and left calf with associated pain and erythema at injection sites. The concern for complicated skin tissue infection was acknowledged and the patient was admitted. The initial urine toxicology report was positive for cocaine, cannabinoids, benzodiazepines, and fentanyl. Upon detailed history-taking, the patient attested to recent changes in the sedative and euphoric effects of fentanyl use, which increased the index of suspicion for xylazine exposure. Immunoassay-based xylazine test strips were positive. Initially, he was administered broad-spectrum intravenous antibiotics, then switched to oral antibiotics at discharge. He was compliant with starting buprenorphine and buprenorphine-naloxone following discharge for management of his severe opioid use disorder. Xylazine's alpha-2-adrenergic agonistic properties in the periphery are thought to initiate a cascade of effects starting with vasoconstriction causing impaired blood perfusion hindering wound healing and increasing vulnerability to secondary infections. This case report aims to alert the medical community about the alarming occurrence of xylazine as an adulterant in illicit drugs and to describe the characteristic skin lesions associated with xylazine injections. Awareness of xylazine use should be considered in patients who develop necrotic skin ulcerations at injection sites along with alterations in typical effects of drug use.

## Introduction

Xylazine, commonly referred to as “Tranq,” is an FDA-approved sedative and tranquilizer solely for veterinary use [1,2]. However, its use as an adulterant in various street drugs, including fentanyl, has been on the rise, leading to its street name, "Tranq-Dope". It is speculated that, due to its cheaper cost compared to fentanyl as well as its enhancing addictive effects, street drug manufacturers are using xylazine to lower costs and increase demand [3].

Besides the sedative effects of xylazine that mimic the effects of opiates, the injections are known to produce distinctive skin ulceration with accompanying necrosis, which can be considered virtually pathognomonic. With a six-fold increase in xylazine presence in fentanyl-associated overdoses between 2019 and 2022 from 4% to 24%, it is of the utmost importance to be mindful of the current street drugs in circulation [4]. This case report aims to alert the community, particularly in South Florida, United States, about the local presence of xylazine adulterants in illicit street drugs, such as fentanyl.

This article was previously presented as a poster at the 2023 American College of Physicians Florida Chapter Annual Scientific Meeting on October 28, 2023.

## Case presentation

**Figure 1:**
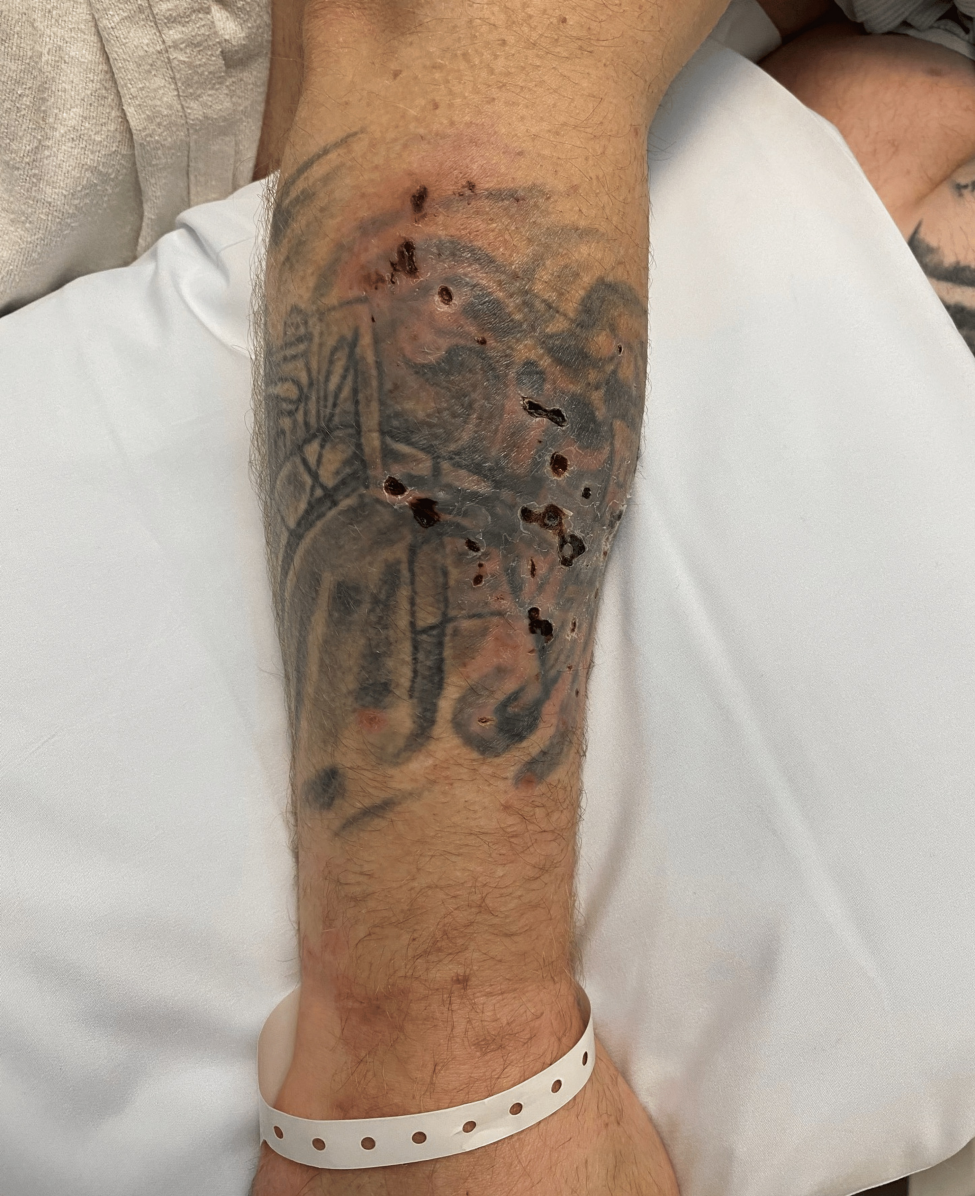
Xylazine-induced necrotic skin ulcerations along the right posterolateral forearm with associated erythema.

**Figure 2:**
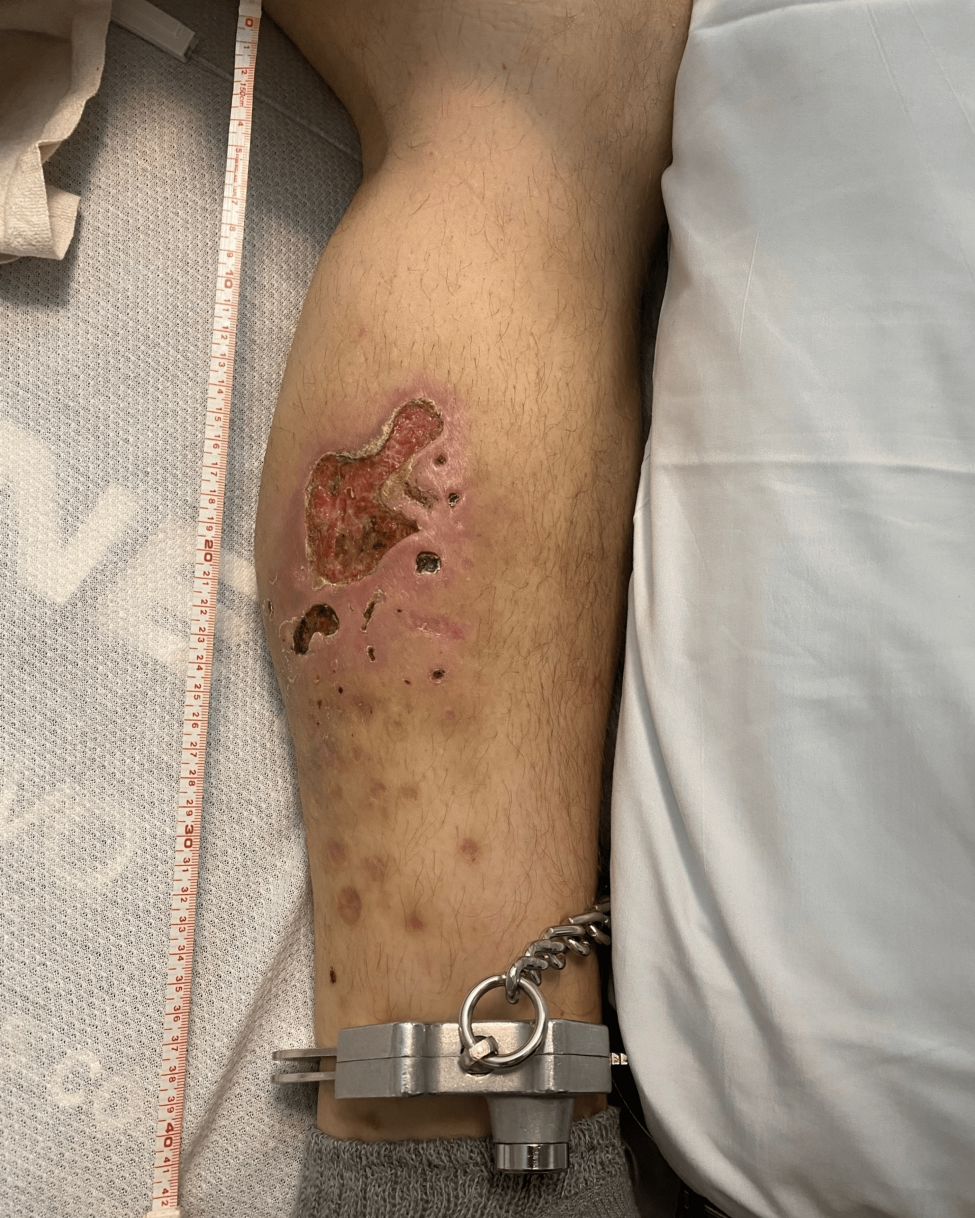
Xylazine-induced necrotic skin ulcerations along the left posteromedial lower leg with associated erythema.

A 41-year-old male in police custody presented to the emergency department (ED) with severe necrotic ulcers on his right posterolateral forearm as well as his left posteromedial lower leg (Figures 1-2). He noted associated pain and erythema around the areas of ulceration. Three weeks before his ED presentation, he denied any skin ulceration at the sites which is where he would typically inject fentanyl, despite a self-reported 20-year history of opioid use disorder. Additional past medical history includes stimulant use disorder and chronic hepatitis C infection. At the time, he was not taking any prescription medications and had no known drug allergies. He noted a family history of hypertension and diabetes with his father who unfortunately passed away due to a myocardial infarction. 

On the physical exam, he was alert and oriented to person, place, and time. Vitals upon ED admission showed that he was slightly hypertensive at 132/83 mmHg and afebrile. His initial laboratory workup was positive for mild anemia, and slight elevations in lactic acid, C-reactive protein (CRP), and aspartate aminotransferase (AST). All other parameters of his comprehensive metabolic panel (CMP) were within normal limits. Imaging of the right upper extremity and left lower extremity was ordered by way of ultrasound, X-ray, and CT scan with all three imaging modalities showing signs of soft tissue edema correlating to cellulitis. Blood cultures identified gram-variable bacilli, which helped explain the elevations of lactic acid and CRP. An initial urine toxicology was positive for cocaine, cannabinoids, benzodiazepines, and fentanyl. 

The patient was ultimately admitted to the floor with concerns of cellulitis and bacteremia superimposed to these skin ulcerations on his arm and leg. He was started on intravenous (IV) vancomycin and cefepime, and a subsequent wound consult was ordered. Upon further history-taking, the patient stated that his “dope felt different than usual” and it “knocked him out more” than prior usage. This history raises an interesting point of how patients may be unaware that they are introducing xylazine into their bodies unintentionally and how xylazine has quietly adultered local street drugs in South Florida. Given this recent history, the index of suspicion of possible xylazine exposure was raised and point-of-care urine analysis for xylazine was ordered, which resulted in a positive result.

During his inpatient stay, his IV antibiotic regimen was altered to discontinue cefepime, continue vancomycin, and switch to bactrim following discharge. Additionally, the patient was compliant with starting buprenorphine while inpatient and starting suboxone following discharge. Over three days, both the patient's pain and erythema had improved with this current regimen, and the patient was discharged to police custody. 

## Discussion

The primary mechanism of action of xylazine involves its role as an alpha-2-adrenergic receptor agonist, which causes its intoxicating effects [1,2,5,6]. Given its mechanism of action, xylazine intoxication presents with typical central nervous system depressing effects such as ataxia, dizziness, drowsiness, dysarthria, hypotension, bradycardia, respiratory depression, and miosis [6]. The onset of these effects can be within minutes and last for eight hours or longer. Duration of effects typically depends on the dosage of xylazine, the route administered, and if used with other drugs such as opioids [6,7]. With the great degree of symptom overlap, it may be difficult to differentiate xylazine intoxication from opioid intoxication. It is important to note that, unlike opioids, the sedative effects of xylazine cannot be reversed by opioid antagonist medications such as naloxone. 

Regarding the specific cause of skin necrosis and ulcer formation induced by xylazine, the exact mechanism remains hypothetical. It is believed that upon injection, xylazine's alpha-2-adrenergic properties trigger vasoconstriction, leading to reduced perfusion, impaired wound healing, and increased susceptibility to secondary infections [5,7]. Furthermore, it is noteworthy that skin necrosis can manifest not only at the site of injection but also in distant areas of the skin [1]. This occurrence of skin necrosis in remote regions adds complexity to understanding the underlying factors involved.

The use of xylazine is progressively becoming more abundant in the United States and is being found in the illicit drug supply. Analysis of Miami-Dade County Medical Examiner Case Data has reported a total of 170 cases of accidental drug overdose deaths in South Florida between 2015 and 2022, and xylazine was listed as the actual cause of death in nearly 40% of these 170 deaths [4]. The FDA has urged healthcare providers to be aware of the possibility of xylazine in patients with polysubstance abuse, especially since there are no current medications approved by the FDA for the management of xylazine withdrawal [8]. The consequences of IV injection use of xylazine produce distinctive skin ulceration with accompanying necrosis, which can be considered virtually pathognomonic.

## Conclusions

The goal of this case report is to alert the community about the presence of xylazine adulterants with street drugs such as fentanyl. Severe xylazine-induced skin ulcers can lead to a plethora of presentations, including epidermal/subcutaneous necrosis and osteomyelitis, which could necessitate limb amputation. Awareness and concern for xylazine use should be considered in patients who develop necrotic skin ulcerations in addition to opioid withdrawal symptoms that cannot be managed with conventional opioid therapies. Further investigations should be directed to high-quality trials researching a potential reversing agent to xylazine as well as epidemiological studies to determine the prevalence of xylazine as an adulterant in the illicit drug supply.
